# Royal Medical and Chirurgical Society

**Published:** 1897-03-13

**Authors:** 


### ROYAL MEDICAL AND CHIRURGICAL SOCIETY.

A paper was read on Tuesday last by Mr. Herbert E. Durham on the "Clinical Bearings of some Experiments on Peritoneal Infections." Issaeff had shown, in 1894, that several different substances, when injected into the peritoneal cavity of guinea-pigs, were capable of affording protection to the animals against an inoculation with cholera vibrios performed on the following day. By means of the preliminary injections, an increase of the "power of resistance of the part" was established, so that an otherwise fatal dose of microbes could be tolerated. This condition was attained in about twenty-four hours, and lasted about four days. It was important to observe that this condition of exalted general resistance had nothing to do with specific or special immunity; an animal protected by a previous injection of simple saline solution being rendered refractory to cholera and other vibrios. It also differed in its transient nature, specific immunity being more intense and lasting. The protection so obtained seemed to be due to the increased bactericidal power of the peritoneal fluid and to the increase in the number of phagocytes in it, produced by the injection of the various substances experimented with; but it was clear that when the substance used for injection was serum immunised to the microbe employed, there was an additional protection due to its own specific or antitoxic properties. In view of these facts, Mr. Durham thought it ought to be earnestly considered whether some preliminary treatment should not be undertaken before operating through the peritoneal cavity upon abscesses which were sure, in the process of being opened, to give rise to a certain risk of peritoneal infection. If this were granted it would become a matter of considerable importance to be able to foretell the sort of microbic infection which was likely to occur in different classes of cases, so as to know the nature of the injection which it would be most profitable to employ in the particular case; the point to be aimed at being to produce an ample leucocytosis in the peritoneal fluid, and also, if possible, to give a superadded specific protection by aseptic means. He had made a number of observations upon fatal cases of peritonitis which had occurred from time to time in Guy's Hospital. In some of the more virulent cases of peritoneal infection the signs of inflammation were but slight, and in their investigation it was of especial importance to make an examination of the lymph tracks which led up into the anterior mediastinum. It was only when animals died of relatively subacute peritoneal infection that pus, flakes of cells, pseudo-membranes, and the like were to be found. As to the bacillus coli, even when it existed in considerable quantities, cocci of various sorts were also present, and must be regarded as important infecting agents even in poly-infections of the peritoneum, so that everything pointed to the desirability of the prophylactic use of anti-streptococcal serum the day before an operation was undertaken in dangerous conditions.

